# Siderophores for molecular imaging applications

**DOI:** 10.1007/s40336-016-0211-x

**Published:** 2016-10-11

**Authors:** Milos Petrik, Chuangyan Zhai, Hubertus Haas, Clemens Decristoforo

**Affiliations:** 1 Faculty of Medicine and Dentistry, Institute of Molecular and Translational Medicine, Palacky University, Olomouc, Czech Republic; 2 Universitätsklinik für Nuklearmedizin, Medizinische Universität Innsbruck, Anichstr. 35, 6020 Innsbruck, Austria; 3 Department of Experimental Nuclear Medicine, Guangzhou Medical University, Guangzhou, Guangdong China; 4 Division of Molecular Biology, Biocenter, Medical University Innsbruck, Innsbruck, Austria

**Keywords:** Siderophores, Desferrioxamine, Triacetylfusarinine C, Infection, Bifunctional chelator

## Abstract

This review covers publications on siderophores applied for molecular imaging applications, mainly for radionuclide-based imaging. Siderophores are low molecular weight chelators produced by bacteria and fungi to scavenge essential iron. Research on these molecules has a continuing history over the past 50 years. Many biomedical applications have been developed, most prominently the use of the siderophore desferrioxamine (DFO) to tackle iron overload related diseases. Recent research described the upregulation of siderophore production and transport systems during infection. Replacing iron in siderophores by radionuclides, the most prominent Ga-68 for PET, opens approaches for targeted imaging of infection; the proof of principle has been reported for fungal infections using ^68^Ga-triacetylfusarinine C (TAFC). Additionally, fluorescent siderophores and therapeutic conjugates have been described and may be translated to optical imaging and theranostic applications. Siderophores have also been applied as bifunctional chelators, initially DFO as chelator for Ga-67 and more recently for Zr-89 where it has become the standard chelator in Immuno-PET. Improved DFO constructs and bifunctional chelators based on cyclic siderophores have recently been developed for Ga-68 and Zr-89 and show promising properties for radiopharmaceutical development in PET. A huge potential from basic biomedical research on siderophores still awaits to be utilized for clinical and translational imaging.

## Introduction

Progress in Molecular Imaging applications in particular in the context of radionuclide-based technologies is dependent on highly specific tracers aiming at an increasing number of available molecular targets. The development of radiopharmaceuticals is impressively advancing based on the progress in radiopharmaceutical chemistry embracing the increasing understanding of the molecular basis of pathophysiology in many clinical fields. Radiometals have been an essential part in this development, initially driven by technetium-99m based radiopharmaceutical developments, today overtaken by the interest in positron emission tomography (PET) with the implementation of gallium-68 in clinical routine and other radiometals entering the arena including zirconium-89, copper-64, scandium-44 and others. Integration of radiometals in “biomolecules” requires the attachment of a chelator binding the metal with high stability without impairing affinity to the target. Nature has designed specific chelators for a variety of metals; an important group is the so-called siderophores (from Greek translating to “Iron-Carrier”) for binding ferric ions, produced by bacteria, fungi and plants. This review summarizes applications of siderophores as chelators for general molecular imaging applications and in particular in the field of infection imaging.

## Methods

**Fig. 1:**
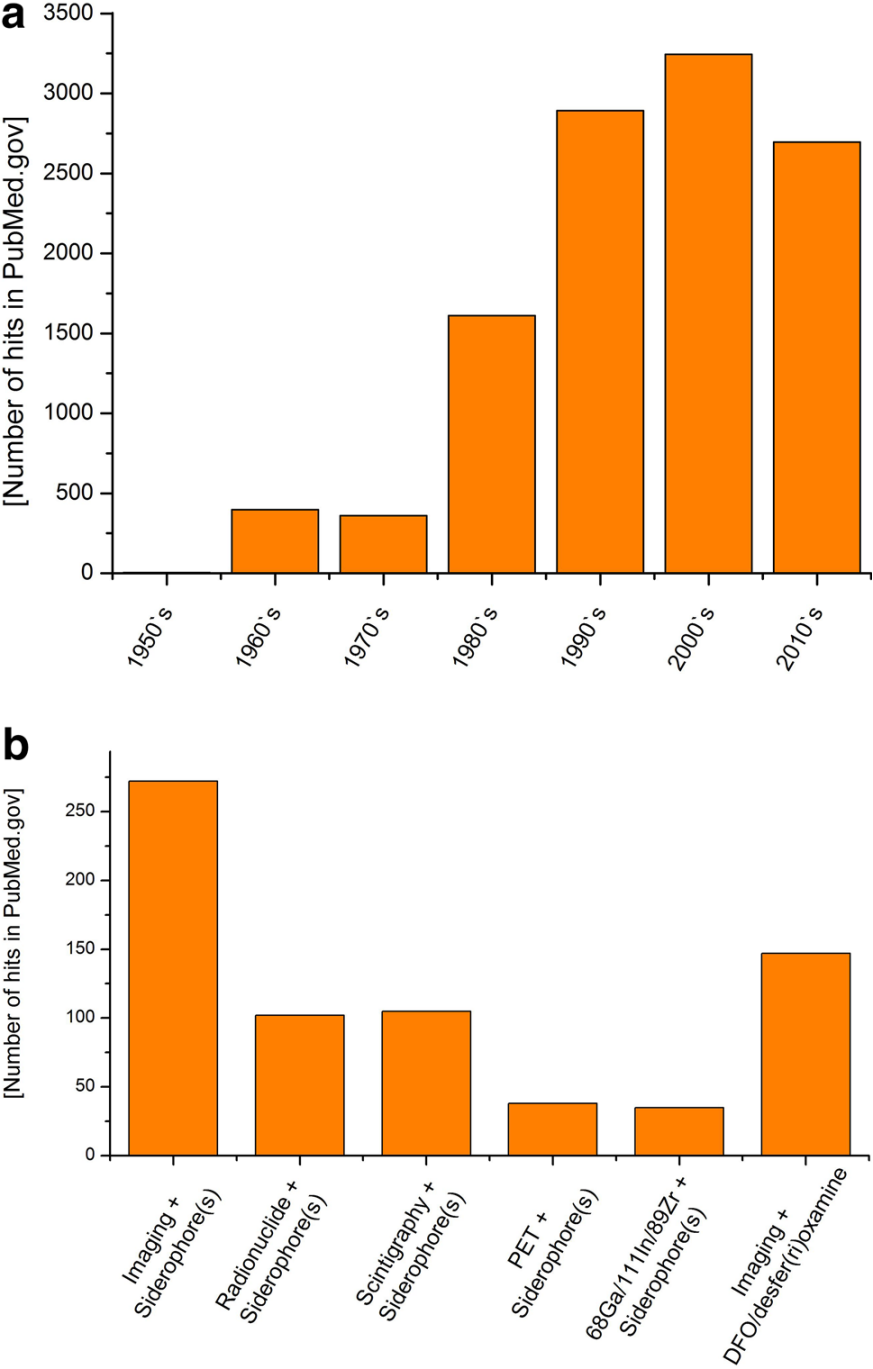
Interest in siderophores in Biomedical Research over the last 60 years based on PubMed-listed publications; **a** search term using “Siderophore” presented in hits/decade; **b** publication hits combining search terms “Siderophore” with various Imaging key words

Siderophores have been very widely investigated in biomedical research. A systematic search in PubMed was carried out, taking into account publications until August 2016. The search term “Siderophore” reveals 11,205 hits in PubMed (August 2016), starting from 1953 with first publications on Mycobactin [1]. Figure 1a shows the distribution of publication over the last 60 years indicating the constant interest of the scientific community in siderophores in biomedical research including preclinical and clinical applications. Combining the search term “Siderophore(s)” or the most widely used siderophore “Desferrioxamine” with key words related to imaging such as “Imaging”, “Radionuclide”, “PET”, “scintigraphy” or specific radionuclides all together 699 publications were found with relations of siderophores to imaging applications (Fig. 1b). Even though systematic search was carried out, the high number of publications made a selection of recent, up to date reviews on the general topic of siderophores or on ^89^Zr labelling based on siderophores necessary. This review also did not intend to analyse the clinical applications or outcomes; meta-analysis or risk assessment was, therefore, not applied.

## Microbial siderophores

**Fig. 2:**
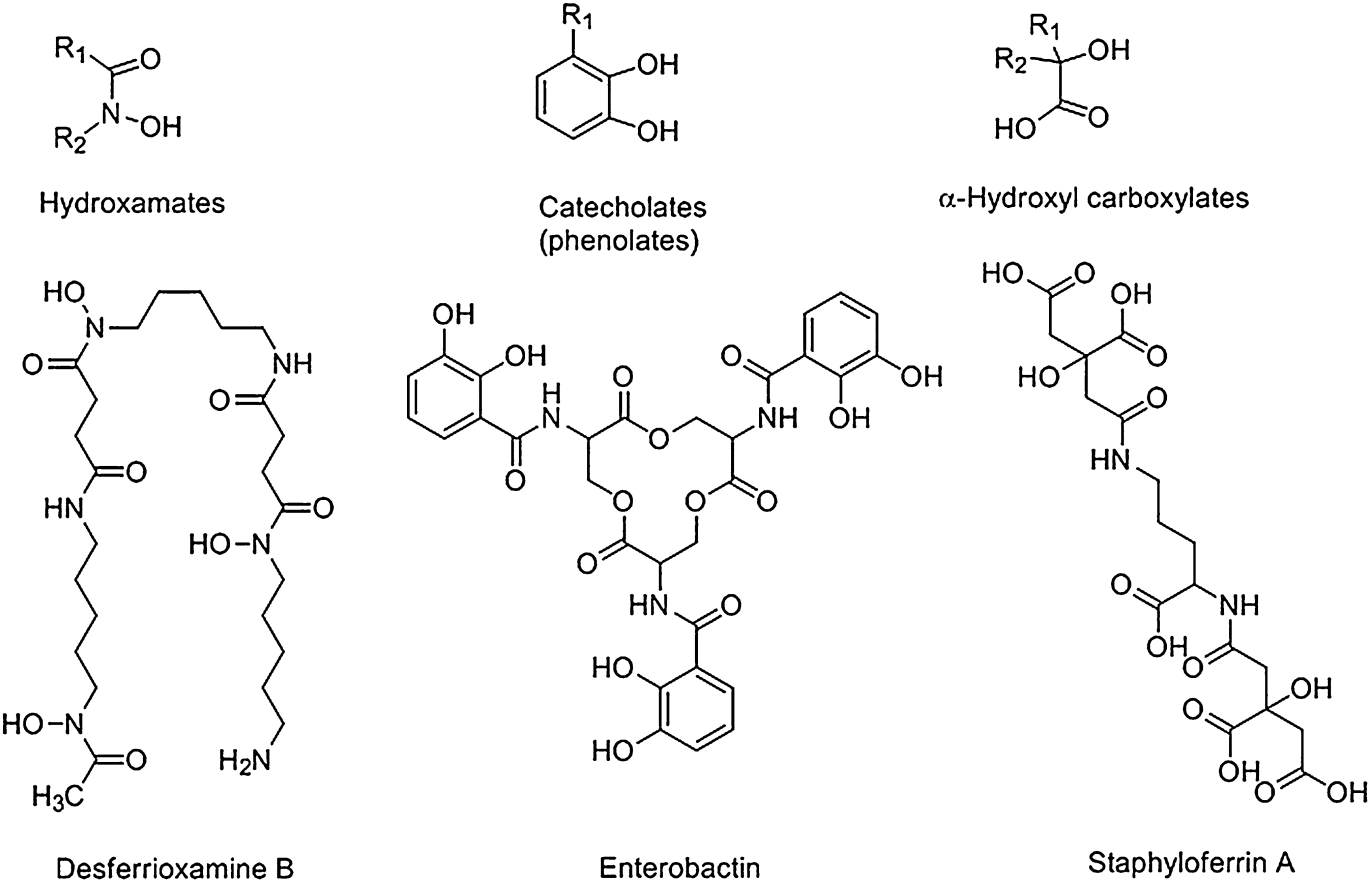
Basic ferric-coordination units (*top*) and examples of natural siderophores (*bottom*)

Iron is an essential cofactor for a variety of cellular processes in all eukaryotes and most prokaryotes including respiration, amino acid metabolism, and biosynthesis of DNA and sterols. Despite its high abundance in the Earth’s crust, the bioavailability of iron is extremely low owing to its oxidation by atmospheric oxygen into sparely soluble ferric hydroxides with a solubility of 10^−18^ M at pH 7.0. Moreover, for pathogens in both plant and animal hosts, iron is usually not freely available but tightly sequestered, e.g. in vertebrates associated with proteins such as transferrin, ferritin and haemoglobin. Blocking iron access to invading microbes represents a key pathway in host defence as a component of innate immunity, termed “nutritional immunity” [2, 3]. Consequently, pathogenic as well as non-pathogenic organisms had to evolve sophisticated strategies to ensure iron supply. Microorganisms are believed to lack mechanisms for iron excretion and, therefore, control of iron uptake is considered the major iron homeostatic mechanism. To satisfy the iron need in diverse niches, bacteria and fungi use different iron acquisition mechanisms, which are transcriptionally upregulated during iron limitation: (1) direct ferrous iron (Fe^2+^) uptake, (2) direct ferric iron (Fe^3+^) uptake, (3) siderophore-mediated ferric iron uptake, and (4) uptake and degradation of haeme. Most microbial species employ more than one system in parallel but not all species use all four strategies. With few exceptions, bacterial and fungal species secrete siderophores to scavenge extracellular iron. Siderophores, low molecular mass (≤1 kD), ferric iron-specific chelators, display a remarkable species-specific, structural diversity with >500 different siderophores being identified [4, 5]. Some bacteria possess plasma membrane-localized siderophores, e.g. mycobactins of *mycobacteria*. In contrast to bacteria, most fungi also possess intracellular siderophores for intracellular transport and storage of iron. Siderophores contain the most efficient iron-binding ligand types in nature, consisting of hydroxamate, catecholate or α-hydroxy-carboxylate ligands (Fig. 2). The most efficient siderophores form hexadentate complexes, satisfying the six co-ordination sites on ferric ions allowing iron-binding constants of 10^20^–10^50^. Examples are enterobactin in the catecholate class, triacetylfusarinine C (TAFC), ferrioxamines (FOX) E and G, as well as the ferrichromes in the hydroxamate class and staphyloferrin in the α-hydroxy-carboxylate class (Fig. 2) [6]. The majority of e.g. fungal siderophores belong to the hydroxamate class. Fungal hydroxamate siderophores can be grouped into four structural families: fusarinines, coprogens, ferrichromes and rhodotorulic acid [6]. The hydroxamate group is built by acylation of the non-proteinogenic amino acid N^5^-hydroxy-l-ornithine, which is derived by hydroxylation of l-ornithine, with acetyl or more complex groups such as anhydromevalonyl. Most fungal siderophores include three of these moieties linked by ester or peptide bonds to form the most efficient hexadentate structures. Cyclization of the siderophore is found in ferrichromes and some fusarinines. Although linear hexadentate siderophores are found in all siderophore classes, there is a tendency for cyclization, thereby enhancing complex and chemical stability.

Siderophore metabolism is highly specific to microbes: Siderophore production involves enzymes that are found exclusively in bacteria and fungi, e.g. nonribosomal peptide synthetases, and siderophore uptake is mediated by specific transporters. In bacteria and fungi, siderophore uptake is mediated by different transport systems. For example, in gram-negative bacteria siderophores have to cross both the outer membrane and the plasma membrane; e.g. ferrichrome type siderophores are transported through the outer membrane via the receptor FhuA, which is energized by the plasma membrane-localized TonB complex, and transported across the plasma membrane via ABC-transporter-dependent movement [7]. In contrast, cellular uptake of siderophore-iron complexes in fungi is mediated by “siderophore-iron transporters” (SITs), which belong to a subfamily of the major facilitator protein superfamily [8]. SITs act most likely as proton symporters energized by the plasma membrane potential. SIT-mediated iron uptake is universally conserved in the fungal kingdom, even in species not producing siderophores such as *Saccharomyces cerevisiae*, *Candida* spp. and *Cryptococcus neoformans* [9]. Moreover, most bacterial and fungal species are able to utilize not only the endogenous siderophores but also siderophore types that are produced by other bacterial or fungal species (so-called xenosiderophores).

**Fig. 3:**
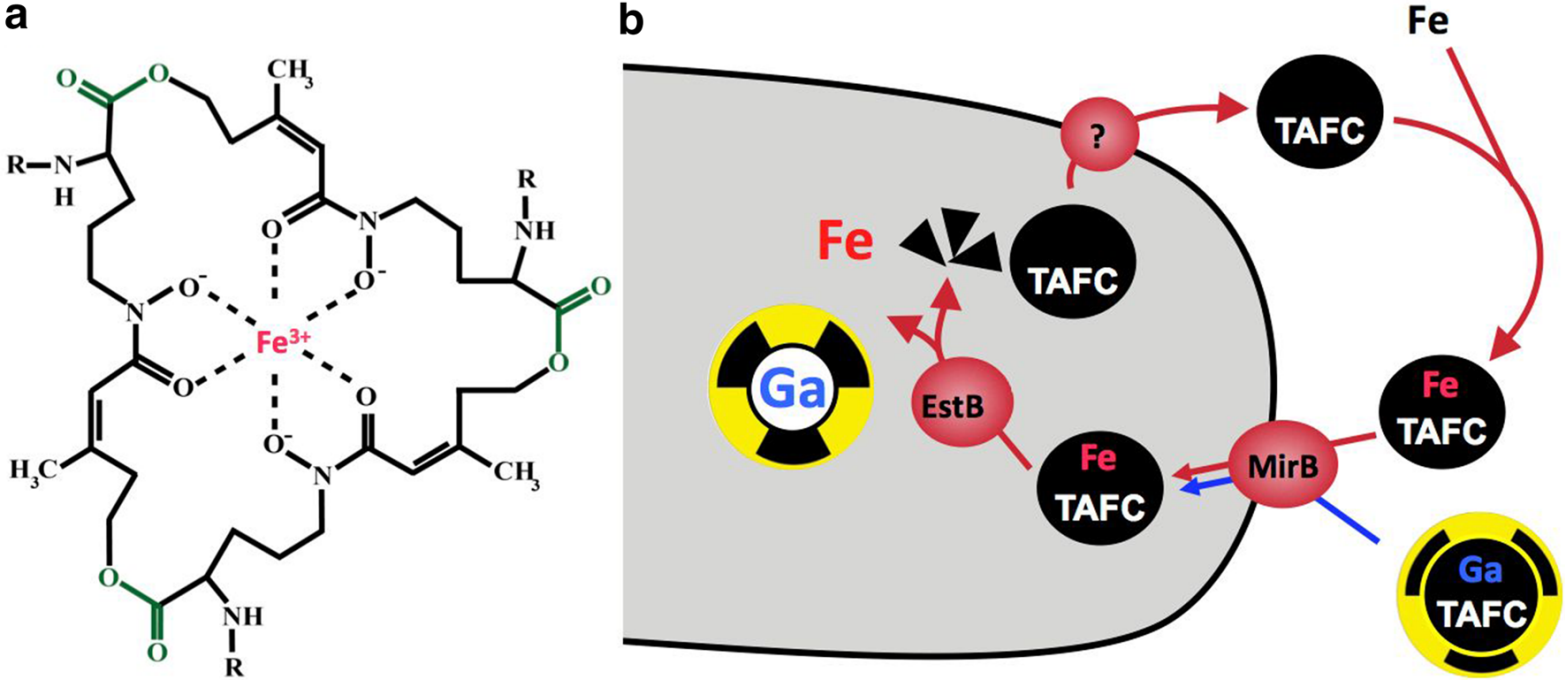
Siderophore-mediated iron uptake in the mold *A. fumigatus*. **a** The cyclic trihydroxamate siderophores FSC (R = H) and TAFC (R = acetyl) are shown in the ferri-form; for TAFC-based nuclear imaging, the iron (shaded in *red*) is replaced by ^68^Ga. **b** TAFC-mediated uptake of iron and gallium into fungal hyphae. TAFC is secreted by an unidentified exporter and the iron/gallium-siderophore complex is taken up by the siderophore transporter MirB. Within the cell, iron release from the siderophore is facilitated by TAFC hydrolysis by the esterase EstB [5]

Taken together, both siderophore biosynthesis and their specific cellular uptake are confined to the bacterial and fungal kingdoms. Moreover, there is overwhelming evidence that the siderophore system is active during infection; e.g. (1) siderophore biosynthesis and uptake are transcriptionally upregulated during iron starvation in vitro as well as in vivo in a murine model for pulmonary infection with the mold *Aspergillus fumigatus* (*A. f.*) [10, 11], and (2) genetic inactivation of siderophore biosynthesis attenuates virulence of *A. f.* in a murine infection model, which demonstrates that siderophore-mediated iron assimilation plays the major role for virulence [12, 13]. Moreover, the siderophore of *A. f.*, triacetylfusarinine C (TAFC) was shown to be able to extract iron from host transferrin [14]. A scheme of TAFC-mediated iron uptake is shown in Fig. 3. Similarly, siderophore biosynthesis was shown to be crucial for the virulence of numerous bacterial species including, e.g. *Yersinia pestis*, *Mycobacterium tuberculosis* or *Pseudomonas aeruginosa* [e.g. 15]. As a result, siderophores were suggested as biomarkers in aspergillosis and tuberculosis [16, 17]. Due to the function of siderophores as virulence determinants, mammals evolved siderophore sequestering proteins, termed siderocalins, and pathogens evolved mechanisms to avoid recognition of their siderophores by siderocalins [18].

Unequivocally, siderophores play a profound role in iron acquisition of most microorganisms. Nevertheless, there are evidences that siderophores can chelate also other metals with physiological relevance, e.g. the siderophore yersiniabactin was recently found to sequester extracellular copper to protect uropathogenic *Escherichia coli* from copper toxicity during human infection [19], while some siderophores appear to be involved in uptake of various non-iron metals such as yersiniabactin in zinc uptake by Yersina pestis [20, 21]. Due to the indispensability of siderophore-mediated iron acquisition, this system is hijacked during microbial competition, e.g. the outer membrane ferrichrome-type siderophore receptor of *E. coli* serves also as receptor for various bacteriophages [22] and naturally evolved siderophore-antibiotic conjugates, termed sideromycins, in which a bactericidal warhead is attached to a siderophore moiety [20, 21]. For instance, albomycins comprise a hydroxamate siderophore unit, reminiscent of those found in fungal ferrichromes, and bactericidal unit that inhibits seryl-tRNA synthetase. Albomycins display a broad-spectrum of antibiotic activity again both Gram-negative and Gram-positive bacteria because of the widespread nature of ferrichrome receptors. These natural “Trojan horses” inspired the development of designed synthetic conjugates [23]. Similarly, gallium salts have been described as potential anti-infectives. In this case, gallium is bound to siderophores and taken up by the pathogen via the siderophore transport system which negatively interferes as iron analogue with the pathogens’s iron homeostasis [24]. A human application of siderophores, which is not related to infectious diseases, is the use of desferrioxamine, a siderophore produced by *Streptomycetes* spp, in treatment of iron overload such as thalassemia to mobilize and decrease body iron stores [25].

## Siderophores for molecular imaging of infection

The accurate localization and characterization of infection and its distinction from inflammation have emerged as one of the greatest challenges of modern medicine. Identification of patients at high risk and early and accurate diagnosis remains crucial for their successful therapy and underlines the urgent need for specific and sensitive diagnostic tools. Molecular imaging methods hold the potential to provide a more robust, non-invasive, selective and sensitive diagnosis of infections leading to improved clinical decisions and a fundamental change in patient management with better healthcare outcomes [26]. Radiological imaging techniques such as computed tomography (CT), magnetic resonance imaging (MRI) and ultrasonography (US) are widely used in clinical practice for identification of infection, although they have major limitations in specificity [27]. Optical imaging represents an interesting future approach to molecular imaging of infection, but no optical probes have been licensed for routine use in the clinic for microbial detection [28]. By contrast, nuclear imaging techniques including PET and SPECT have a rich history of different radiolabelled probes (radiopharmaceuticals) for imaging of infectious processes in patients. These include ^111^In- or ^99m^Tc-labelled leucocytes, ^99m^Tc-anti-granulocyte antibody, ^99m^Tc-diphosphonates in the context of bone scanning, ^67^Ga-citrate and 2-[^18^F]-fluorodeoxyglucose [26]. These probes target predominantly secondary effects of infection such as increased blood flow and vascular permeability, activated endothelial cells or polymorphonuclear cell migration limiting their specificity or have other shortcomings related to blood manipulation or induction of immune response (HAMA) [29, 30]. Even though new developments are emerging especially for bacterial infections such as radiolabelled antimicrobial peptides [26], nuclear medicine clinicians are still awaiting improved radiopharmaceuticals overcoming these limitations.

An interesting group of molecules, which could fulfill the requirements on the ‘optimal imaging agent’ for molecular imaging of infections, appears to be (radio)labelled siderophores. Table 1 summarizes applications of siderophores as imaging agents. They can be prepared either by the introduction of appropriate radiometal to the natural (iron-)siderophore complex via the exchange of iron or artificially by the modification of natural siderophore with a chromophore suitable for optical imaging [31–34]. Already in the 1970s and 1980s, first investigations of radiolabelled siderophores, including desferrioxamine (DFO), were already reported with gamma-emitting radionuclides—^67^Ga and ^111^In [35–38]. Gallium is an isosteric diamagnetic substitute for Fe(III) [39] and, thus, the affinity constants of many siderophores for gallium are in the range of their iron counterparts. At that time, it was also demonstrated that under reducing conditions, Ga(III) can rapidly displace Fe(III) from siderophores, whereas without concerted reduction of the iron no significant exchange was observed [40]. Emery and Hoffer [41] have used ^67^Ga to study the uptake mechanisms for different siderophores in *Ustilago sphaerogena* and found this energy-dependent process to be indistinguishable from that of its Fe(III) counterpart. They even postulated an involvement of siderophore binding in the accumulation of ^67^Ga-citrate in inflammatory lesions. A number of investigations were made with ^3^H, ^14^C, ^55^Fe and ^59^Fe labelled siderophores mainly to study iron transport or siderophore uptake mechanisms in microorganisms or plants [e.g. 42–44] unsuitable for molecular imaging and, therefore, cannot be used for detection of microbial infections in vivo. By contrast, radionuclides used in the studies of Moerlein and Emery [37, 40, 41]—^67^Ga and ^111^In—have found widespread use in nuclear medicine for SPECT imaging. Over the past decade, PET has experienced a significant increase applying a variety of positron emitting radiometals [45]. Recently, ^68^Ga use in particular is showing a dramatic growth because of the applicability in labelling of diverse range of compounds and because it is obtained from a long shelf-life and relatively inexpensive ^68^Ge/^68^Ga generator system [46].

**Table 1:** Applications of siderophores as imaging agents

Imaging modality	Type of label	Siderophore	Application	Reference
SPECT	^67^Ga, ^111^In	Desferrioxamine	Development of novel radiopharmaceuticals; tumor and abscess imaging	[35, 36, 38]
SPECT	^67^Ga, ^111^In	Enterobactin	Ligand for radiopharmaceuticals	[37]
SPECT	^67^Ga	Ferrichrome, ferrichrome A, rhodotorulic acid, triacetylfusarinine C, malonichrome, desferrioxamine	Microbial iron transport	[40, 51]
PET	^68^Ga, ^89^Zr	Ferrichrome, ferrichrome A, triacetylfusarinine C, desferrioxamine, desferrioxamine E, coprogen, fusarinine C, ferricrocin	Infection imaging	[47–54]
Fluorescence imaging	Rhodamine B analogue, anthracene analogue, 7-nitrobenz-2-oxa-1,3-diazole analogues, fluorescein analogues	Ferrichrome, desferrioxamine, pyochelin	Microbial iron uptake and transport; siderophore/iron metabolism	[31–33, 55–58]

**Fig. 4:**
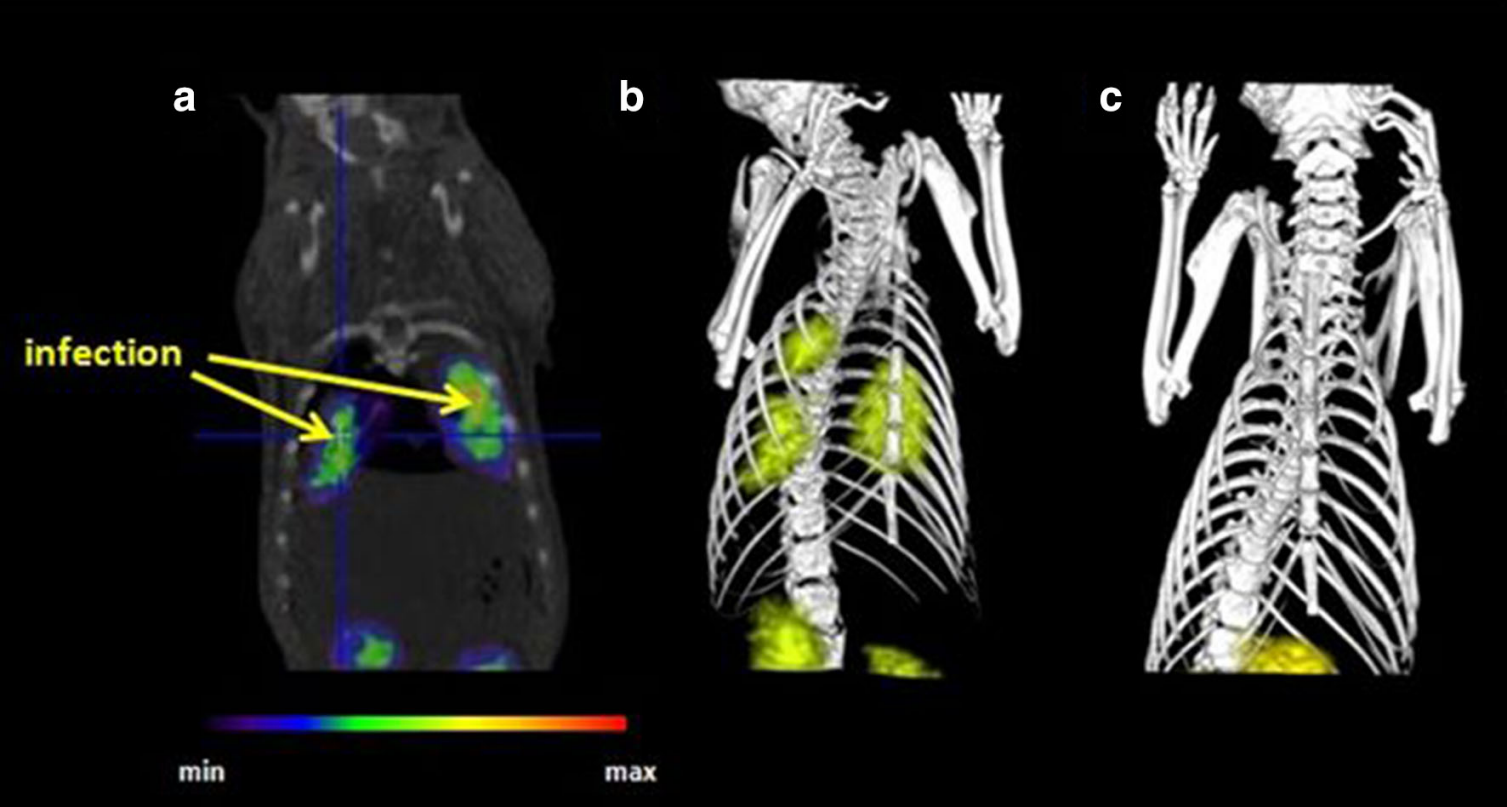
Micro-PET/CT (Albira PET/SPECT/CT small animal imaging system, Bruker Biospin Corporation, Woodbridge, CT, USA) imaging of *A. fumigatus* [coronal slices (**a**) and 3D images (**b**)] in a rat infection model and non-infected rat (**c)** 45 min post-intravenous injection of ^68^Ga-TAFC showing clear accumulation in infected [(**a**) and (**b**)] and no accumulation in healthy **c** lung tissue

More than 30 years after the first attempts of labelling siderophores with ^67^Ga [36–38, 40, 41], we evaluated the use of ^68^Ga labelled siderophores for PET imaging of fungal infections [47]. In proof of concept studies, which should confirm or refute the possibility of PET imaging of infections caused by *Aspergillus fumigatus* (*A. f.*) using ^68^Ga-siderophores [48], it was demonstrated that desferrisiderophores, particularly triacetylfusarinine C (TAFC), can be easily radiolabelled with ^68^Ga using a few micrograms of the siderophore and exhibit high chemical stability. Uptake of ^68^Ga-TAFC by *A. f.* was upregulated under iron starvation conditions and could be blocked with an excess of siderophore or NaN_3_, indicating specific and energy-dependent uptake. A variety of different siderophores such as fusarinine C (FSC), TAFC, coprogen, various ferrichrome and ferrioxamine-type-siderophores displayed excellent ^68^Ga-radiolabeling properties [49]. However, only ^68^Ga-TAFC and ^68^Ga-ferrioxamine E (FOXE), a siderophore produced by *Streptomycetes*, displayed a good combination of fungal uptake in culture, suitable pharmacokinetics for imaging (i.e. rapid clearance from organs and circulation with predominant renal excretion) and, in particular, excellent metabolic stability [50]. Significantly different in vivo behaviour compared to ^68^Ga-citrate (i.e. non-specific infection and inflammation PET imaging agent) was also found [51]. High contrast imaging of *A. f.* pulmonary infection in a rat model was achieved using micro-PET/CT technology [50, 52], exhibiting pronounced accumulation of ^68^Ga-TAFC in infected areas extremely early after onset of infection, which increased with severity of infection and correlated with abnormal CT images (Fig. 4). Significant accumulation of ^68^Ga TAFC was found neither in sterile inflammations nor in tumour cells [53], which also have a high iron metabolism. We also investigated the uptake of ^68^Ga-TAFC in a number of different fungal and bacterial species, which revealed high specificity for *Aspergillus* species, with no significant uptake by *Candida* and bacterial species, in particular. By comparison, FOXE displayed high in vitro uptake by *Staphylococcus aureus*, which was surprisingly not confirmed in vivo [53]. An interesting exception among *Aspergillus* species is *Aspergillus terreus*, which lacks the ability to take up TAFC but accumulates FOXE. Besides the investigations with siderophores labelled with ^68^Ga, we have also attempted to radiolabel siderophores with different radionuclides. So far we have succeeded to label TAFC, FOXE, desferrichrome A (FCHA) and DFO with zirconium-89 [54]. The interest in ^89^Zr has increased over the last years as it displays almost ideal properties allowing imaging of biological processes at late time points after the tracer application. Even though ^89^Zr has comparably low positron abundance and due to the long half-life (78.4 h) results in higher radiation dose, it allows long-term follow-up especially of slowly accumulating biomolecules such as antibodies, nanoparticles and other large biomolecules both for preclinical and clinical applications, thereby complementing ^68^Ga with its limitations of a very short half-life (67.7 min). Comparing the in vitro and in vivo characteristics of ^68^Ga-siderophores with their ^89^Zr counterparts, we found analogous properties with the potential for longitudinal *Aspergillus* infection imaging [54]. From all these studies, we concluded that ^68^Ga-labelled siderophores, in particular ^68^Ga-TAFC, have a high potential to be used as radiopharmaceuticals to specifically image *Aspergillus* infections in patients.

Overall radiolabelled Siderophores certainly have the potential to be a highly specific tool for infection imaging, considering the essential role of the siderophore system for iron acquisition and virulence of microorganisms together with its upregulation during infection, whereas they are not utilized by mammals. This is also related to the low toxicity of siderophores exemplified by DFO, which is used safely in close to gram amounts for iron overload disease repeatedly. Selecting appropriate siderophores can also lead to a high specificity for particular microorganisms, e.g. being able to distinguish between certain fungal and bacterial infections. The requirement for upregulation of the siderophore transporters to accumulate the radiolabelled siderophore, however, will require a rather acute status of infection; therefore, it can be expected that its main role can be envisaged in a rather acute setting, such as detection and specific characterization of invasive Aspergillosis, with its live-threatening consequences rather than in a more chronic or less aggressive infection setting. This can only be revealed in a clinical setting; therefore, the first clinical studies of ^68^Ga-siderophores are currently eagerly awaited.

Besides radiolabelling, also other attempts have been made to use siderophores for pathogen detection. Several groups have developed strategies of synthesizing siderophore-chromophore conjugates for optical imaging [31–33, 55–58]. Siderophores (e.g. ferrichromes, pyochelin and DFO) derivatized with various fluorescent probes, such as fluorescein, rhodamine, 7-nitrobenz-2-oxa-1,3-diazole and anthracene, were used for the monitoring of siderophore transport in different microorganisms including bacteria (e.g. *Pseudomonas* spp.) [31, 32, 58] and fungi (*Ustilago maydis*, *Saccharomyces cerevisiae*, *Candida albicans* and *Rhizzopus arrhizus*) [33, 55, 57]. The microbial activity was not altered by the attachment of various functionalities and fluorescent siderophore analogues became invaluable tools in the investigation of molecular mechanisms involved in microbial iron transport and acquisition. Accordingly, these artificial siderophore analogues could also serve as a tool for in vivo diagnostic imaging or targeting of microbial pathogens [34].

The recognition of the role of siderophores as important microbial iron transporters has led to the exploitation of this pathway in a ‘Trojan Horse’ strategy not only for pathogen detection, but also for the development of therapeutic strategies [34, 59]. Banin et al. [60] have used siderophore-metal complex combining a strong siderophore, DFO with non-radioactive gallium for the treatment of *Pseudomonas aeruginosa* (*P.a.*) infection. The Ga-DFO complex was designed as an antioxidant that acts by ‘push and pull’ mechanism, sequestering ferric ions (the siderophore effect) and, in turn, releasing gallium ions that further compete with ferric ions at iron-binding sites of proteins. The Ga-DFO served as a ‘Trojan Horse’ that interferes with iron metabolism and delivers toxic gallium to *P.a.* cells. The antimicrobial effect of Ga-DFO to *P.a.* infections showed promising results; nevertheless, it warrants further investigation. Moreover, a number of studies on complex siderophore-drug conjugates have been made to test their potential as effective antimicrobial agents [23, 34, 61–63]. It could be speculated that these siderophore conjugates could be radiolabelled or derivatized and used for molecular imaging of infections.

## Siderophores as bifunctional chelators

Table 2 summarizes applications of siderophores as bifunctional chelators, combining the two functions of metal coordination with the coupling property to a targeting vector.

**Table 2:** Applications of siderophores as bifunctional chelators

Radionuclide	Siderophore	Radiopharmaceutical	References
^67^Ga	DFO	Albumin	[65]
^67^Ga	DFO	Fibrinogen	[66, 67]
^67^Ga, ^111^In	DFO	Antibodies	[69–73]
^68^Ga	DFO	Nanobodies	[74]
^67^Ga	DFO	Folate	[75]
^67^/^68^Ga	DFO	Octreotide	[76–78]
^89^Zr	DFO	Antibodies	[82–84]
^89^Zr	DFO	RGD peptides	[91]
^89^Zr	DFO	Nanoparticles, carbon nanotubes, microspheres	[92–94]
^89^Zr	DFO	Nanocolloids	[95]
^89^Zr	DFO	Proteins	[96, 97]
^89^Zr	DFO*	Bombesins	[100]
^68^Ga	FSC	Peptide multimers	[101, 102, 104]
^89^Zr	FSC	Peptide multimers	[103]

### Desferrioxamine and gallium

Already early in the development of targeted radiopharmaceuticals, siderophores were considered as chelators for radiometals. Initial studies focussed on gallium-67 as a gamma-emitting isotope with a half-life of 78.3 h for planar scintigraphy and SPECT imaging. ^67^Ga-Citrate was introduced for tumour studies and due to its similarities with Fe^3+^ DFO was proposed to enhance tumour to blood ratios in tumour imaging [64]. Three hydroxamate groups of DFO coordinate Ga^3+^ with fast kinetics and high affinity, forming a stable 1:1 chelate with high radiochemical yield. The free amino group can be used as coupling side to bioactive molecules. Already in 1982, the proof of principle was shown by coupling DFO to albumin for binding ^67^Ga, proposing DFO as bifunctional chelating agent [65]. A glutaraldehyde coupling reaction was applied and the authors showed a superior in vivo stability of ^67^Ga-DFO-HSA over ^131^I-labelled HSA and provided first images in patients. A first targeted application was reported in the same year by coupling DFO to fibrinogen [66]. A large number of DFO molecules were introduced to human fibrinogen using dialdehyde starch (DAS) as a spacer-functional polymer. Increased accumulation of ^67^Ga-fibrinogen in venous thrombi was depicted at 48 h after injection about 60 % of patients [67]. Other applications of DFO-conjugated macromolecules followed soon with radiolabelled lectins [68], which failed in tumour detection. A more successful approach was the development of DFO-conjugated monoclonal antibodies and antibody fragments. Motta-Hennessy C et al. [69] established conditions for the coupling of DFO with the bifunctional reagent glutaraldehyde to two rat IgG2b monoclonal antibodies M10/76 and 11/160, specific for the Hooded rat sarcomata MC 24 and HSN, respectively, which maintained their capacity for binding to their tumour-associated antigens. Koizumi et al. [70] compared the homocoupling reagent glutaraldehyde with two other heterocoupling reagents, *N*-succinimidyl-3-(2-pyridyldi-thio)propionate and succinimidyl-6-maleimidohexanoate, linking desferrioxamine to antibodies through alkylamine, disulphide, and thioether bonds, and showed superiority of thioether bonds in terms of tumour targeting and pharmacokinetics. Bartal et al. [71] compared the labelling of MAb 23H7, binding to human sarcoma, with ^67^Ga using glutaraldehyde-coupled DFO and ^111^In via DTPA, whereby higher specific activities were achieved with ^67^Ga. Amino-dextran-DFO was used to derivatise an anti-melanoma monoclonal antibody (TP41) for labelling with In-111 with promising results especially reduced liver uptake [72]. DFO as bifunctional chelator for antibodies was also proposed for radiotherapeutic applications using ^67^Ga Auger electrons. Govindan et al. [73] prepared different DFO-antibody conjugates and reported two main problems limiting further development. First, the stability was inadequate for the 3-day half-life of the nuclide. Second, the labels were poorly retained within cells after Ab internalization and catabolism. More recently, a novel bifunctional chelate (BFC) *p*-isothiocyanatobenzyl-DFO (Df-Bz-NCS), originally developed for ^89^Zr labelling, was used to prepare anti-EGF Nanobody conjugates of DFO for ^68^Ga labelling for PET applications [74]. Fast radiolabelling, high tumour uptake and tumour to normal tissue ratios in nude mice bearing A431 xenografts were obtained with the fast kinetics of the ^68^Ga-Nanobody conjugates, indicating a promising application of DFO conjugates with ^68^Ga.

Besides proteins also smaller molecules were conjugated to DFO for radiolabelling with ^67/68^Ga. Folic acid was covalently linked to DFO via an amide bond using a simple carbodiimide coupling reaction. ^67^Ga-DF-folate(gamma) exhibited specific uptake and was proposed as a diagnostic agent for noninvasive imaging of folate receptor-positing tumours [75]. ^67/68^Ga-DFO-Octreotide (SDZ 216-927), comprising DFO coupled to octreotide via a succinyl linker [76, 77], showed specific uptake in Somatostatin receptor expressing tumour models and was proposed as PET imaging agent. However, in patients ^67^Ga DFO-Octreotide radioactivity was detectable in the circulation even after 24 h; the blood clearance curve was much slower than the one of OctreoScan (^111^In-DTPA-Octreotide) due to relatively high protein binding in human serum [78]. So, overall a number of attempts have been made to develop siderophore-bioconjugates based on DFO for radiolabelling with ^67/68^Ga and to a limited extent with ^111^In, however, with inconclusive results in particular related to its stability especially at high specific activities [79], thereby being replaced mainly by aminocarboxylate-based chelators such as DOTA or NOTA.

### Desferrioxamine and zirconium-89

In contrast to that in the past decade, DFO has established its role in the context of ^89^Zr-labelling [80–84]. ^89^Zr was proposed as a diagnostic radionuclide for quantitating the biodistribution of radiolabelled antibodies. The high affinity of zirconium for hydroxamic acid groups makes DFO a suitable and effective chelator for Zr^4+^. Meijs and co-workers initially reported that DFO exhibits rapid and efficient labelling with a 1:1 ratio of metal to chelate and demonstrates good stability with regard to demetallation, releasing less than 0.2 % of the metal in serum after 24 h [85]. Further evaluation of the complex by Holland and co-workers utilizing density functional theory (DFT) models exhibited Zr-DFO as an octadentate complex combining the six binding oxygens of DFO with two additional water molecules. Also, stability studies over longer periods of time indicated that still less than 2 % demetallation occurs after 7 days in serum [86].

The first clinical trial with an ^89^Zr-labelled antibody revealed the low immunogenicity of the DFO-conjugate [87] allowing repeated applications of the DFO immunoconjugate. For the coupling of DFO to antibodies, most widely 2,3,5,6 tetrafluorophenyl TFP-activated ester of *N*-succinyl-DFO-Fe forming stable amide bonds with free amines have been applied [88], or alternatively *p*-isothiocyanato-DFO forming a stable thiourea bond with lysine residues [89]. Standardized protocols have been established [90] making ^89^Zr labelling for Immuno-PET applications ever more widely applicable. Several reviews have summarized the latest progress of ^89^Zr-DFO-conjugated antibodies [82–84].

The use of ^89^Zr-labelled bioactive molecules using siderophores is not limited to the antibodies. Beyond antibodies, ^89^Zr-DFO conjugated to peptides and peptide multimers [91], nanoparticles [92, 93], carbon nanotubes [94], Albumin nanocolloids [95], and proteins [96, 97] has also been investigated.

### Improvement of DFO for ^89^Zr

Despite the prevalent use of ^89^Zr-DFO-conjugated antibodies for preclinical studies and clinical applications, several preclinical studies reported bone accumulation of dissociated ^89^Zr ranging from 3 to 15 % after 3–7 days [86, 98, 99]. This insufficient stability of the ^89^Zr-DFO complexes is attributed to the incomplete coordination of ^89^Zr^4+^ by DFO and the linear structure of DFO. Based on the knowledge of DFO, Patra et al. developed an octadentate DFO analogue termed DFO*, which fully saturates the coordination sphere of Zr^4+^, by coupling an additional hydroxamic acid entity to DFO [100]. DFT calculations predicted the expected molecular structure involving coordination through the eight oxygen atoms of all four hydroxamic acid moieties. Coupling the model peptide bombesin ([Nle14]BBS(7–14)), DFO*-bombesin showed a remarkably improved stability in comparison to the DFO analogue when challenged with 300- to 3000-fold molar excess DFO over the course of 1 day. The in vitro experiment demonstrated that the new chelator did not influence the properties of the peptidic vector. Based on those results, DFO* holds promise to provide new PET imaging agents with superior stability profiles; applications on DFO* coupled antibodies are awaited soon.

### Other siderophores as bifunctional chelators

**Fig. 5:**
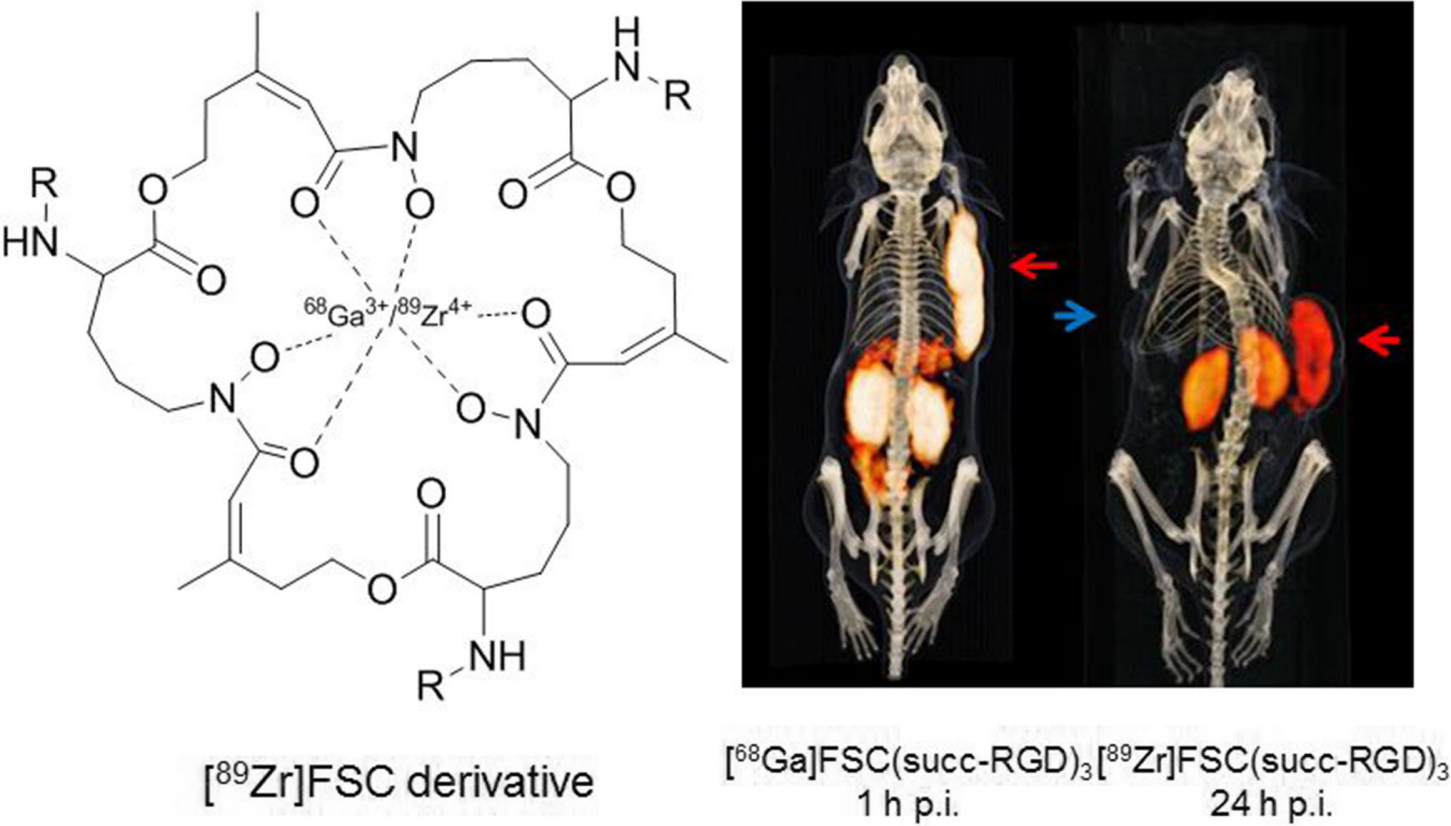
Structure of the siderophore FSC as bifunctional chelator for ^68^Ga and ^89^Zr, three-dimensional volume projections of fused microPET/CT images of M21/M21-L tumor xenograft bearing nude mouse ([^68^Ga]FSC(succ-RGD)_3_ at 1 h, [^89^Zr]FSC(succ-RGD)_3_5 MBq) at 24 h p.i. *Red arrow* αvβ3 integrin-positive M21 tumor; *blue arrow* αvβ3 integrin-negative M21-L tumor (from [103, 104])

Recently, we reported that Fusarinine C (FSC), a representative of the class of hydroxamate siderophores, is a promising ^68^Ga and ^89^Zr bifunctional chelator [101–104]. FSC, possessing three hydroxamic acid groups for binding ^68^Ga or ^89^Zr similar to DFO embedding an additional cyclic structure, offers a potential advantage with respect to the stability of ^68^Ga/^89^Zr complexes. FSC not only allows fast and highly selective labelling with ^68^Ga in a wide pH range and results in high specific activities, but also shows very high stability of ^68^Ga-FSC complexes at low concentration demonstrating the superiority over DFO which was reported to be unstable at low ligand concentrations (<50 nM) [79]. Compared to ^89^Zr-DFO, ^89^Zr-FSC derivatives showed excellent in vitro stability and resistance against transchelation in phosphate-buffered saline (PBS), ethylenediaminetetraacetic acid solution (EDTA) and human serum for up to 7 days making it an alternative as ^89^Zr BFC [103]. The three primary amines of FSC facilitate the derivatization of FSC with targeting biomolecules in a number of ways, also applying the concept of multivalency. By attaching a cyclic RGD peptide, binding to integrin α_v_β_3_ expressed during angiogenesis, via a succinic acid linker (FSC-(RGD)_3_), high stability ^68^Ga complexes with excellent receptor-binding properties and in vivo targeting were prepared (Fig. 5), superior to monomeric [^68^Ga]NODAGA-RGD [104]. Currently, monovalency- and divalency FSC for ^68^Ga/^89^Zr labelling are under investigation and different coupling strategies e.g. click chemistry are being investigated.

## Conclusion

Extensive publications from the last decades have described a wide variety of Fe^3+^ binding siderophores produced by bacteria and fungi. Their role in iron acquisition and human diseases has been reported and methods for chemical modification, chemical synthesis and even radiolabelling with a variety of radiometals are available. This knowledge has been translated towards radiopharmaceuticals for molecular imaging in general and specific imaging of infection in particular. There are many opportunities to further use this knowledge towards development of new, improved radiopharmaceuticals for molecular imaging in PET, but also towards theranostics and optical imaging applications.
